# Fellowships, Grants, & Awards

**Published:** 2007-05

## Global Research Initiative Program, Behavioral/Social Sciences (R01)

This program is intended to promote productive development of foreign investigators from low- to middle-income countries, trained in the United States or in their home countries through specific Fogarty International Center D43 or U2R training programs, to independent researcher in their home countries or other low- or middle-income countries. This program will dovetail with several specific research training mechanisms, including training in the NIH Intramural Visiting Fellows Program, the Fogarty International Center research training programs (D43 or U2R), the NIDA INVEST, or Humphrey Fellowships, NIEHS junior scientist programs, and the Human Frontier Science Program, as part of a broader program to enhance the scientific research infrastructure in low- to middle-income countries, to stimulate research on a wide variety of high-priority health-related issues of importance in those countries, and to advance NIH efforts to address health issues of global import.

The specific goal of this initiative is to provide funding opportunities for the increasing pool of foreign social and behavioral scientists, clinical investigators, nurses, and other health professionals, on their return to their home countries, with state-of-the-art knowledge of research methods to advance the critical issues in global health through social and behavioral sciences research.

After their term of research training, low- to middle-income country participants supported by this announcement are expected to continue independent and productive scientific careers, including providing expert training and consultation to others, and/or research on behavioral and/or social science issues within their home institutions.

This announcement contributes to the FIC mission and the broad NIH initiative to reduce health disparities among nations by strengthening research infrastructure in low- to middle-income countries, particularly those with the least economic resources. It also provides the opportunity for recently trained international health and health care researchers to continue their projects after returning home.

Definitions of “behavioral” and “social”: For the purposes of this initiative, the term “behavioral” refers to overt actions; to underlying psychological processes such as cognitions, emotion, temperament, and motivation; and to biobehavioral interactions. The term “social” encompasses sociocultural, socioeconomic, and sociodemographic status; to biosocial interactions; and to the various levels of social context from small groups to complex cultural systems and societal influences.

The core areas of behavioral and social sciences research are those that have a major and explicit focus on the understanding of behavior and social processes, or on the use of these processes to predict or influence health outcomes or health risk factors. These core areas of research are divided into basic (or fundamental) research and clinical research.

As part of its global health initiative under the Department of Health and Human Services (DHHS), the Fogarty International Center (FIC), in partnership with the National Eye Institute (NEI); the National Institute on Drug Abuse (NIDA); the National Institute of Environmental Health Sciences (NIEHS); the National Institute of General Medical Sciences (NIGMS); all of the National Institutes of Health (NIH), and the Office of Behavioral and Social Sciences Research (OBSSR), the Office of Dietary Supplements (ODS), and the Office of Research on Women’s Health (ORWH), all of the Office of the Director (OD) of the NIH, invites applications from current and former NIH-supported foreign research trainees to compete for funds that will support their research efforts upon return to their home countries. To be eligible, foreign scientists must meet at least one of the following criteria: 1) at least 2 years of research training experience under an FIC-supported training grant (classified by the D43 and U2R mechanisms); 2) 1 year of such D43 or U2R training experience coupled with 1 year of significant, well-documented, mentored research experience (e.g., through an NIH research award such as the NIAID Small Research (R03) Grant (IRID-NIAID) program); 3) 1 year of the NIDA INVEST or Humphrey Fellowship (http://www.drugabuse.gov/International/HHHRF.html) plus at least one additional year of significant, well-documented, mentored research; 4) at least 2 years of research training experience through the NIH Intramural Visiting Fellows Program; 5) 1 year of training through an F05 international fellowship program and 1 subsequent year of mentored research; 6) recipients of Long-Term Fellowship awards through the Human Frontier Science Program, who come from low- and middle-income countries, and who have spent at least 2 years in research training; 7) at least 1 year of training in the United States and one additional year of significant, well-documented, mentored research, in the United States or abroad, leading to a completed master’s degree or doctoral degree, at least partially funded through a Fogarty International Center research training program, with preapproval by the program director; 8) foreign trainee researchers from low- or middle-income countries trained under NIEHS R01, R37, and P01 programs, as described at http://www.niehs.nihgov/dert/programs/capacity.htm.

All such training and research must either have been done in the United States or have been part of in-country research associated with a degree or mentored postdoctoral research under the D43 or U2R award mechanism and completed within 4 years of the receipt date of this announcement.

Candidates who are more than 5 years beyond their training, but who have interrupted their careers because of illness or family commitments, may also apply. They must clearly explain the interrupted hiatus and must clearly demonstrate the potential for productive independent research.

Current NIH Visiting Fellows are encouraged to apply in a timely fashion, i.e., as they begin their preparation to return home. They, as all applicants, may apply within 4 years of completing training.

Through various programs, the NIH has made a significant investment in training biomedical and behavioral researchers. For example, the NIH Visiting Fellows Program currently hosts more than 1,600 junior scientists from almost 100 countries for periods of 1–5 years. In addition, the NIH D43 research training and capacity building and the U2R cooperative agreement grant mechanisms allow hundreds of foreign researchers to receive training at prominent institutions in both the United States and their home countries in a range of biomedical and behavioral research areas critical to advancing global health. In summary, training supported by NIH is critical to these young investigators as they develop independent research careers.

As junior scientists complete training programs in the United States and in their home institutions under FIC D43 or U2R programs, many find it difficult to secure the support needed to continue their research projects and careers as independent researchers in their home countries. This Global Research Initiative Program (GRIP) provides the opportunity for junior foreign scientists to compete for such funds through a peer-reviewed process. This is a critical adjunct in the continuation of promising independent research careers that will be of benefit to the investigators’ home countries and the world at large. Women and underrepresented minority scientists in their countries are especially encouraged to apply for these re-entry grants. Project proposals should be geared toward the research interests of the applicant and the focus on high-priority health and health care problems in the investigator’s home country that also carry global importance, and are of interest to the collaborating NIH Institutes, Centers, and Offices (ICs) listed on the first page of this announcement, as well.

It is expected that research topics will vary. Specific research interests of partnering ICs can be found on the ICs’ websites, as listed in the beginning of this announcement. Research related to women’s health, including studies of gender differences in disease onset and progression, identification of behavioral strategies that are effective in encouraging healthy lifestyles in young girls and women, as well as behavioral strategies to encourage prevention of diseases such as STDs and diseases with higher prevalence among women (including infectious diseases, lupus, multiple sclerosis, and depression), are particularly encouraged. Research on healthy outcomes of pregnancy and child survival, and population research as associated with both behavioral and social, and economic research is encouraged. Research related to the health effects of human exposures to environmental agents is encouraged. Research focused on behavioral and social determinants and their effects on health is also encouraged. All research must be performed in accordance with NIH and U.S. government regulations regarding the responsible conduct of research. This announcement precludes the support of research involving enrollment in pilot studies for clinical trials, or the actual support of clinical trials since the resources and infrastructure to support and oversee such trials generally exceed the resources available under this award mechanism. Applicants are encouraged to visit the website of the Office of Human Research Protection (OHRP) (http://www.hhs.gov/ohrp/) that outlines these regulations. For information on animal protection in research, see http://grants.nih.gov/grants/olaw/olaw.htm.

This announcement contributes to the FIC mission and to the broad NIH initiative to reduce the health disparities among nations by strengthening research infrastructure in developing countries, particularly those with the least economic resources. Additionally, it provides the opportunity for recently trained international health and health care researchers to continue their projects after returning home.

Evaluation of the program will occur on an ongoing basis. Because this is a program to move research trainees to the status of independent investigator, there are several outcomes to be measured: 1) development of the laboratory capabilities or research projects; 2) training of other potential researchers; 3) publications in international peer-reviewed journals, as well as local journals; 4) participation in workshops, seminars, and international conferences; 5) collaborations with past mentors, as well as other U.S., international, or local researchers; and 6) attraction of funding from other sources.

As part of its assessment of the impact and scientific productivity of this program, FIC plans to track researchers and their trainees for at least 10 years after beginning their independent research. Evaluation may focus on the success of researchers (as measured by the number and quality of publications, presentations, courses, awards, subsequent employment, etc.), their sustained commitment to research careers, their ability to attract funding for their work, their contributions to future international collaborations, their influence on the development of scientific research in their countries, and their ability to act as consultants, teachers, and role models to other local investigators and further disseminate the lessons learned. Applicants should describe potential metrics that would indicate the success of the individual researcher and the success in capacity building at the home institution, including the impact of the program on research at the institution in the home countries of researchers and their trainees.

This Funding Opportunity Announcement (FOA) will use the NIH Research Project Grant (R01) award mechanism.

The applicant will be solely responsible for planning, directing, and executing the proposed project.

This FOA uses “Just-in-Time” information concepts. It also uses the nonmodular budget formats (see http://grants.nih.gov/grants/funding/modular/modular.htm).

All foreign applicants must complete and submit budget requests using the Research & Related Budget component found in the application package for this FOA. See NOT-OD-06-096, 23 August 2006.

This award is nonrenewable. An investigator cannot receive a second award in this program.

Applicants must download the SF424 (R&R) application forms and the SF424 (R&R) Application Guide for this FOA through Grants.gov/Apply.

Note: Only the forms package directly attached to a specific FOA can be used. You will not be able to use any other SF424 (R&R) forms (e.g., sample forms, forms from another FOA), although some of the “Attachment” files may be useable for more than one FOA.

For further assistance, contact GrantsInfo, 301-435-0714, (telecommunications for the hearing impaired: TTY 301-451-0088), or by e-mail: GrantsInfo@nih.gov.

The letter of intent dates for this PAR are 21 August 2007, 2008, and 2009 with the application receipt dates 21 September 2007, 22 September 2008, 21 September 2009.

The complete version of this PAR is available at http//www.grants/guide/pa-files/PAR-07-328.html.

The complete list of agency contacts is available at http://grants.nih.gov/grants/guide/pa-files/PAR-07-328.html.

Reference: PAR-07-328.

## Non-Biodefense Emerging Infectious Diseases Research Opportunities (R01)

The goal of this FOA is to increase understanding of the transition of microbes from a nonpathogenic state to a state pathogenic for humans.

One area of interest is to promote research in emerging zoonotic viruses, which will provide predictive tools to anticipate the emergence of new viral pathogens. Many emerging viral diseases are zoonotic in origin; it is estimated that approximately 62% of human infectious diseases are zoonotic and an estimated 70% of newly emerging diseases are zoonotic in origin. The recent emergence and transmission to humans of SARS CoV from civet cats and deadly avian influenza clearly demonstrate that emerging zoonotic viral diseases are a serious public health problem that needs to be understood fully. Although it is clear that one of the principle mechanisms involved in the emergence of new viral diseases is a change in the host range, there are many gaps in our knowledge about the mechanism(s) involved and how zoonotic viruses establish themselves in humans. An understanding of these processes will be necessary to anticipate and prepare for the emergence of new viruses in humans.

Another area of interest is the study of human bacterial pathogens that exist in colonizing, rather than infective states. In colonizing states these human pathogens are in consortia with other microbes, such as when *Staphylococcus aureus* colonizes skin or mucosal tissues without causing disease or when *Clostridium difficile* colonizes the gut without causing illness or when *Neisseria meningitidis* is in the nasopharyngeal tract without causing disease. Little if anything is known about the full complement of microbes present or the structure and functioning of the microbial consortium. It is important to know what microbes are present, in what proportions, and how the consortium functions metabolically. Clearly, the consortium has an interface with the host and this may play a critical role in determining if the microbes remain as colonizers or cause disease. There are several potential colonizing niches in the body for which this concept applies such as skin, gastrointestinal, nasopharyngeal, and genitourinary tracts. Metagenomics as well as microscopic techniques that allow for visualization of the physical structure of the microbial consortium are experimental approaches of interest.

Two recent workshops (Emergence of New Epidemic Viruses through Host Switching and Workshop on Basic Bacterial Research), sponsored in part or in whole by NIAID, resulted in reports that identify areas that are in need of additional research. This FOA is intended to address some of these areas.

Relevant topics of interest include, but are not limited to: 1) Studies on the natural history of emerging viruses, viral reservoirs, natural means of transmission, variation and pathogenesis (excluding HIV and viruses among the NIAID Category A, B & C Priority Pathogens); 2) Studies on the factors that control host–host transmission: pathogenesis studies focused on the development of emerging human pathogenic viruses; viral and host control of viral shedding and infection; 3) Studies on the evolutionary events surrounding the transfer of viruses to new hosts, sequence analysis of viruses before and after the transfer, emergence of variants in the donor host, the transfer event, and post-transfer adaptation; 4) Characterizing human microbial pathogens as they live in nonpathogenic microbial consortia. Research should focus on describing what microbes are present in the consortia, and the metabolism of the consortia, their physical structure and interaction with the environment. Examples of possible experimental approaches include metagenomics, single-cell analysis, and microscopy; 5) Understanding the relationship between microbiota and human health as it influences emergence of disease or new human pathogens; 6) Identifying signals and systems in bacterial communication relevant to the development of pathogenesis or emergence of new human pathogens; 7) Determining whether and how microbiota and human pathogens communicate with host cells and respond to the mammalian immune system; 8) Developing strategies to manipulate chemical signaling of human pathogens within the microbiota.

This FOA will not support studies on HIV or pathogens among the NIAID Category A, B & C Priority Pathogens.

This Funding Opportunity Announcement (FOA) will use the NIH Research Project Grant (R01) award mechanism.

The applicant will be solely responsible for planning, directing, and executing the proposed project.

This FOA uses “Just-in-Time” information concepts. It also uses the modular as well as the nonmodular budget formats (see http://grants.nih.gov/grants/funding/modular/modular.htm).

Specifically, if you are a U.S. organization and are submitting an application with direct costs in each year of $250,000 or less (excluding consortium Facilities and Administrative [F&A] costs), use the PHS398 Modular Budget component provided in the SF424 (R&R) Application Package and SF424 (R&R) Application Guide (see specifically Section 5.4, “Modular Budget Component,” of the Application Guide).

U.S. applicants requesting more than $250,000 in annual direct costs and all foreign applicants must complete and submit budget requests using the Research & Related Budget component found in the application package for this FOA. See NOT-OD-06-096, 23 August 2006.

Applicants must download the SF424 (R&R) application forms and the SF424 (R&R) Application Guide for this FOA through Grants.gov/Apply.

Note: Only the forms package directly attached to a specific FOA can be used. You will not be able to use any other SF424 (R&R) forms (e.g., sample forms, forms from another FOA), although some of the “Attachment” files may be useable for more than one FOA.

For further assistance, contact GrantsInfo, 301-435-0714, (telecommunications for the hearing impaired: TTY 301-451-0088), or by e-mail: GrantsInfo@nih.gov.

The application submission dates of this PA are available at http://grants1.nih.gov/grants/funding/submissionschedule.htm.

The complete version of this PA is available at http://grants.nih.gov/grants/guide/pa-files/PA-07-246.html.

Contacts: N. Kent Peters, Division of Microbiology and Infectious Diseases, National Institute for Allergy and Infectious Diseases, Room 4068, MSC-6603, 6610 Rockledge Drive, Bethesda, MD 20892-6603 USA, 301-496-7728, fax: 301-402-2508, e-mail: petersn@niaid.nih.gov; Eun-Chung Park, Division of Microbiology and Infectious Diseases, National Institute for Allergy and Infectious Diseases, Room 4103, MSC-6604, 6610 Rockledge Drive, Bethesda, MD 20892-6604 USA, 301-402-0071, fax: 301-402-2508, e-mail: epark@niaid.nih.gov; Irene Anne Eckstrand, National Institute of General Medical Sciences, 45 Center Drive, Room 2AS.25K, MSC 6200, Bethesda, MD 20892 USA, 301-594-0943, e-mail: eckstrai@mail.nih.gov.

Reference: PA-07-246.

